# *Origanum vulgare* subsp. *virens* (Hoffmanns. & Link) Bonnier & Layens Essential Oils: Chemotypes and Bioactivity as Antifungal, Antifeeding and Enzyme Inhibitors

**DOI:** 10.3390/plants14193001

**Published:** 2025-09-28

**Authors:** Rui Ferreira, Mariana Martins, Vanessa Santos, Duarte Sardinha, Wilson R. Tavares, Samuel Sabina, Guacimara Espinel, Maria Carmo Barreto, Luísa Oliveira, Raimundo Cabrera, Paula Castilho

**Affiliations:** 1 CQM—Centro de Química da Madeira, Campus da Penteada, Universidade da Madeira, 9020-105 Funchal, Portugal; rui.ferreira@staff.uma.pt (R.F.); 2108020@student.uma.pt (M.M.); vanessasilvasantos259@gmail.com (V.S.); 2 Laboratório de Qualidade Agrícola, Direção de Serviços dos Laboratórios Agrícolas e Agroalimentares, Direção Regional de Agricultura e Desenvolvimento Rural, Secretaria Regional de Agricultura e Desenvolvimento Rural, 9135-372 Camacha, Portugal; duarte.sardinha@madeira.gov.pt; 3 cE3c—Centre for Ecology, Evolution and Environmental Changes, Azorean Biodiversity Group & CHANGE—Global Change and Sustainability Institute, University of the Azores, 9500-321 Ponta Delgada, Portugal; wilson.r.tavares@uac.pt (W.R.T.); maria.cr.barreto@uac.pt (M.C.B.); 4 Unidad de Fitopatología, Sección de Biología, Facultad de Ciencias, Universidad de La Laguna, 38204 La Laguna, Tenerife, Spain; srodrisa@ull.edu.es (S.S.); gespinel@ull.edu.es (G.E.); rcabrera@ull.edu.es (R.C.); 5 CBA—Biotechnology Centre of Azores, Faculty of Sciences and Technology, University of the Azores, 9500-321 Ponta Delgada, Portugal; maria.lm.oliveira@uac.pt

**Keywords:** *Origanum vulgare* subsp. *virens* (Hoffmanns. & Link) Bonnier & Layens, essential oil, antifungal activity, antifeeding activity, acetylcholinesterase, α-glucosidase, β-glucosidase, phytopathogen

## Abstract

Essential oils (EOs) from the leaves of *Origanum vulgare* subsp. *virens* (Hoffmanns. & Link) Bonnier & Layens, representing three chemotypes—thymol-rich, carvacrol-rich, and a mixed thymol–carvacrol type—were chemically characterized and comparatively assessed for their antifungal, insecticidal, and enzyme-inhibitory activities. This integrated approach provides a comparative assessment of all three chemotypes across multiple biological models, including phytopathogenic fungi, insect bioassays, and key enzyme targets. All EOs displayed antifungal activity for the tested phytopathogenic fungi (*Alternaria alternata*, *Botrytis cinerea*, and *Fusarium oxysporum*) at concentrations above 0.5 mg/mL, with the thymol-rich chemotype showing the highest activity. The minimum inhibition concentration for *Oidium farinosum* conidial growth was determined and found to be similar for thymol and carvacrol chemotypes and lower for the terpene mixture. Insect control activity was evaluated by an antifeeding assay, where carvacrol and especially thymol chemotypes can be classified as feeding deterrents. EOs and standards revealed a weak toxicity against *Ceratitis capitata*, with less than 20% mortality at a concentration of 50 mg/mL, and both chemotypes were found to be ineffective in preventing egg deposition. The acetylcholinesterase (AChE) inhibition assay revealed that carvacrol had the greatest inhibitory effect on AChE, followed by EOs, and, finally, thymol. Regarding the α- and β-glucosidase (α- and β-GLU) inhibitory assays, thymol had the strongest inhibitory effect on α-GLU, while plant β-GLU was not inhibited by the standards or OEs.

## 1. Introduction

Phytopathogenic fungi and insects are major threats to crops, causing substantial yield losses and economic damage, with fungi responsible for 70–80% of the estimated 10–15% annual loss in major crops.

Among fungi, *Botrytis cinerea* Pers. and *Alternaria alternata* (Fr.) Keissl affect a broad range of crops, causing pre- and post-harvest diseases such as spots, blights, and gray mold [1,2,3]. *Fusarium oxysporum* Schltdl is another major pathogen that induces vascular wilt in several host plants, as well as having the ability to produce mycotoxins with the potential for neurotoxic effects and disruption of cell metabolism in animals [4,5,6]. Obligate biotrophic fungi of the genus *Podosphaera*, also known as *Oidium*, are responsible for powdery mildew in more than 10,000 plant hosts, leading to significant productivity losses [7,8].

Among polyphagous insects, *Chrysodeixis chalcites* Esper (golden twin-spot moth), is a significant horticultural pest across Southern Europe, the Middle East, and Africa [9]. The five larval instars of this insect are particularly destructive, with a feeding behavior that causes severe defoliation of crops [10]. Modesto Del Pino et al. [11] reported a shift in feeding behavior, with larvae beginning to feed on banana fruits instead of leaves, thereby rendering the fruit commercially unpleasant and leading to an increase in treatment costs. The mediterranean fruit fly, *Ceratitis capitata* Wied, is a highly adaptable species, native to sub-Saharan Africa and with worldwide distribution [12]. This species poses a significant threat to the fruit industry, ranking among the most destructive pests as its larvae feed and develop on many deciduous, subtropical, and tropical fruits, and some vegetables [13,14]. *C. capitata* can serve as a potential vector for the bacterial pathogen *Erwinia amylovora*, responsible for fire blight, which mainly affect plants in the *Rosaceae* (rose) family as well as fruit trees such as apples and pears [15,16].

Conventional control strategies largely rely on organophosphate-based pesticides; however, concerns regarding environmental pollution, resistance, and regulatory restrictions have driven interest in alternative approaches, including integrated pest management [17]. This trend focuses on exploring new botanical biopesticides derived from agricultural and forestry by-products or underutilized plants rich in secondary metabolites, such as flavonoids, hydroxycinnamic acids, terpenes, and alkaloids, by evaluating their cytotoxic and phytochemical properties for effectiveness against pests and pathogens that threaten crops and cultures [18,19].

Botanical essential oils (EOs) are promising biopesticides due to their antimicrobial, insecticidal, and enzyme-modulating activities. EOs are complex mixtures characterized by an abundance of terpenes and terpenoids, with aldehydes, ketones, and homologues of phenylpropanoids also present. Most of the biological activity of EOs is associated with oxygenated terpenes, such as alcohols and phenolic terpenes. In some cases, interactions between the diverse classes of phytoconstituents may lead to antagonistic or synergistic effects that contribute to the biological activity of EOs, and even minor components of EOs such as hydrocarbons can play a critical role in these effects [18,20]. Species of *Thymus* and *Origanum* display strong antimicrobial activities because of the presence of phytoconstituents including the isomers thymol and carvacrol [20,21,22]. In addition, *O. vulgare* EOs exhibit anti-acetylcholinesterase (AChE) activity due to carvacrol and/or thymol, their main constituents. Some studies have highlighted the ability of these compounds to inhibit AChE [23,24,25,26].

The aim of this study was to assess and compare biological properties from three different chemotype EOs extracted from distinct *Origanum vulgare* subsp. *virens* (Hoffmanns. & Link) Bonnier & Layens grown in the Macaronesia Islands. Gas chromatography–flame ionization detector (GC–FID) quantification and gas chromatography–mass spectrometry (GC–MS) were used for characterization of bioactive compounds. The antifungal activity was evaluated against obligate parasites (*Erysiphaceae* family) and phytopathogen fungi (*Alternaria alternata*, *Botrytis cinerea*, and *Fusarium oxysporum*). The insecticidal activity was studied on *Ceratitis capitata* (adult mortality and oviposition deterrence) and *Chrysodeixis chalcites* (larval feeding deterrence). To explore the mechanisms of action of EOs and pure compounds, and possible synergistic or antagonistic interactions that could affect their efficacy and potential impacts on non-target organisms, in vitro assays were carried out with neurochemical and digestive enzymes.

We hypothesized that the three major chemotypes of *O. vulgare* subsp. *virens* (thymol-rich, carvacrol-rich, and mixed) would exhibit distinct and complementary biological activities, with thymol- and carvacrol-dominant oils showing different strengths across antifungal, insecticidal, and enzyme inhibition models, while the mixed chemotype may present intermediate or combined effects

## 2. Results and Discussion

### 2.1. Extraction Yield and EO Phytochemical Profile

The extraction yield was similar for the three samples of *Origanum vulgare* subsp. *virens* (Hoffmanns. & Link) Bonnier & Layens, ranging from 1.9% (*v*/*w*) for OVPEF to 2.0% (*v*/*w*) for OVPS and OVLL. The EOs were characterized by a pale yellow color and a distinct odor. The chemical composition of the EOs and the relative percentage of their constituents are summarized in Table 1.

The principal chemical composition for the three chemotypes was determined using both a polar Supelco SPB-PUFA column (GC-FID) and a non-polar Agilent J&W HP-5 column (GC-MS), with retention indices assigned based on comparison with a retention index database and literature. For the polar column, significant RI variations exceeding 10 index units (IU) were observed for several compounds, attributed to slight differences in polarities of polyethylene glycol stationary phases [27,28]. In total, eight major volatile components were identified for each sample, accounting for 90.7% for OVPEF, 85.3% for OVPS and 96.1% for OVLL. The EOs in this study consisted of monoterpenoids (63.0–78.7%); monoterpenes (9.3–28.3%); methyl ether terpenes (0.9–4.6%), and sesquiterpenes, of which only β-caryophyllene was quantified (0.5–1.1%), but a large variety of other sesquiterpenes and sesquiterpene alcohols was detected and accounted for up to 14% of “other” components mentioned in Table 1. ANOVA confirmed that thymol and carvacrol contents differed significantly among the three chemotypes (*p* < 0.05), with thymol predominating in OVPS, carvacrol in OVPEF, and both in similar proportions in OVLL. Significant differences were also observed for γ-terpinene, p-cymene, and carvacrol methyl ether, while α-pinene, α-terpinene, and β-caryophyllene did not differ consistently among chemotypes.

The main component of the EO for OVPEF was carvacrol (73.04% ± 1.07), which was significantly higher (*p* < 0.05) than in OVPS (4.16 ± 0.35%) or OVLL (32.18 ± 1.54%). Thymol in this chemotype (5.65 ± 0.47%) was comparatively low, as was the monoterpenoid biosynthetic precursor γ-terpinene (5.97% ± 0.38).

In contrast, the sample OVPS contained thymol as the major component (59.19 ± 0.90%), which was significantly higher (*p* < 0.05) than in OVPEF (5.65 ± 0.47%) or OVLL (30.22 ± 0.60%). This sample also showed the highest γ-terpinene content (14.81 ± 0.75%), which was significantly greater than in OVPEF (5.97 ± 0.38%) and OVLL (18.77 ± 0.75%) (*p* < 0.05).

**Figure 1:**
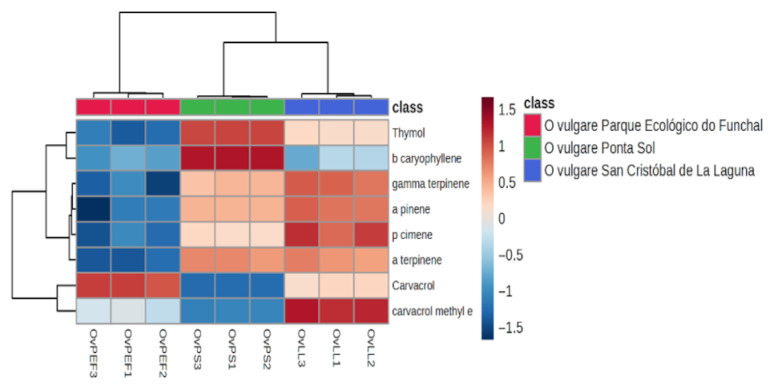
Hierarchical clustering heatmap for the *O. vulgare* subsp. *virens* chemotypes.

The mixed chemotype (OVLL) presented comparable amounts the two phenolic isomers carvacrol (32.18 ± 1.54%) and thymol (30.22 ± 0.60%), and the difference between these two compounds was not statistically significant (*p* > 0.05). However, OVLL contained significantly more p-cymene (5.05 ± 0.30%) than OVPS (3.57 ± 0.21%) (*p* < 0.05), while the difference from OVPEF (2.11 ± 0.29%) was not significant, being grouped in the same cluster as OVPS (Figure 1).

Comparison with the literature confirms that these chemotypes are consistent with previously described *O. vulgare* subsp. *virens* populations from Mediterranean and Macaronesia regions. As noted, the relative abundance of carvacrol and thymol is highly variable, depending on geography, climate, and genetic factors [29,30]. The predominance of monoterpenoids in our samples (63–79%) aligns with earlier findings describing cymyl chemotypes as the dominant group in southern European *O. vulgare* subsp. *virens* populations [31,32,33,34].

Mancini et al. [35] and Lombrea et al. [21] determined that *O. vulgare* subspecies with a EO yield > 2% are characterized by a more active cymyl pathway, favoring the biosynthesis of carvacrol and thymol from their precursors γ-terpinene and p-cymene, with a reverse correlation of abundance most noticeable between carvacrol and γ-terpinene. These findings were challenged by Krause et al. [36], who identified the complete biosynthetic pathway of thymol and carvacrol (Supplementary Materials Figure S1). The precursor γ-terpinene is oxidized by cytochrome P450 monooxygenases from the CYP71D family (most notably CYP71D179-T and CYP71D180-C), resulting in the formation of unstable cyclohexadienol intermediates. These intermediates are further dehydrogenated by a short-chain dehydrogenase/reductase TvSDR1 to generate ketone intermediates that form the aromatic backbone of thymol and carvacrol via keto-enol tautomerism. In the absence of TvSDR1, the dehydration of the cyclohexadienol intermediate leads exclusively to the production of p-cymene. The relative proportions of p-cymene vs. thymol or carvacrol in the volatile constituents are influenced by the enzymatic activities of CYP71D and TvSDR1, and the physical distance between the two proteins in the cell, due to a close association of dehydrogenase—cytochrome P450 leads to lower production of p-cymene. The relatively high levels of p-cymene and carvacrol methyl ether in our mixed chemotype OVLL are consistent with this mechanistic explanation.

Our results agree with the study by Lukas et al. [37], which analyzed 502 *O. vulgare* individuals from various subspecies and identified three main monoterpene chemotypes—cymyl-, sabinyl-, and linalool/linalyl acetate-rich—of which cymyl chemotypes predominated in southern Europe, a trend also reflected in our samples.

### 2.2. Fungicide and Fungistatic Activity

#### 2.2.1. Phytopathogenic Fungi

The three *O. vulgare* subsp. *virens* chemotypes were evaluated against *Botrytis cinerea*, *Fusarium oxysporum*, and *Alternaria alternata* to determine growth inhibition thresholds and inhibitory concentration (IC_50_) and are described in Table 2.

The evaluation of the EOs showed complete mycelium inhibition against all fungi at a concentration of 1 and 0.5 mg/mL. These results suggest a successful antifungal activity at higher concentrations. Subsequently, for the calculation of the concentration of the EO that induces 50% of the mycelium inhibition, these EO were further evaluated against the three phytopathogenic fungi at lower concentrations; namely, 0.5, 0.1, 0.05, and 0.01 mg/mL.

Statistical analysis (ANOVA with post hoc Tukey test, *p* < 0.05) confirmed that thymol-rich chemotype (OVPS) consistently exhibited the strongest antifungal activity, showing lower IC_50_ values against *Alternaria alternata*, *Botrytis cinerea*, and *Fusarium oxysporum* compared with the carvacrol-rich (OVPEF) and mixed (OVLL) EOs. The mixed chemotype OVLL generally displayed intermediate activity, suggesting that the equimolar presence of thymol and carvacrol may dilute the antifungal potency of the dominant monoterpenoids. Although all chemotypes achieved complete inhibition at ≥0.5 mg/mL, activity declined sharply below this threshold, indicating that relatively high concentrations are required for fungicidal effects.

**Figure 2:**
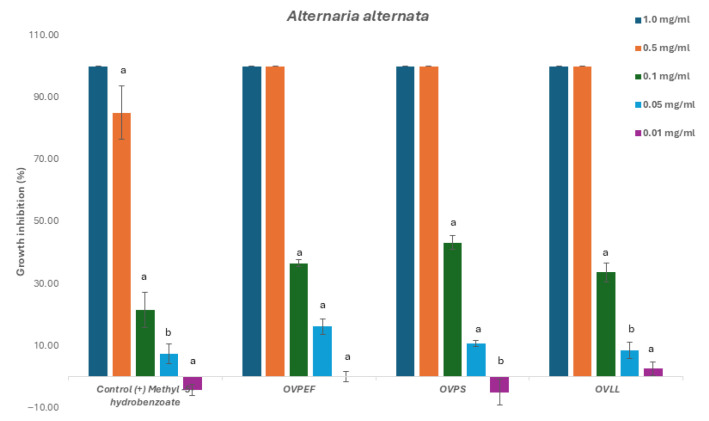
Antifungal effects (% growth inhibition) of the chemotypes of *O. vulgare* subsp. *virens* EOs against *Alternaria alternata.* ^a,b^ Statistically significant differences by ANOVA, Tukey’s test, *p* < 0.05.

**Figure 3:**
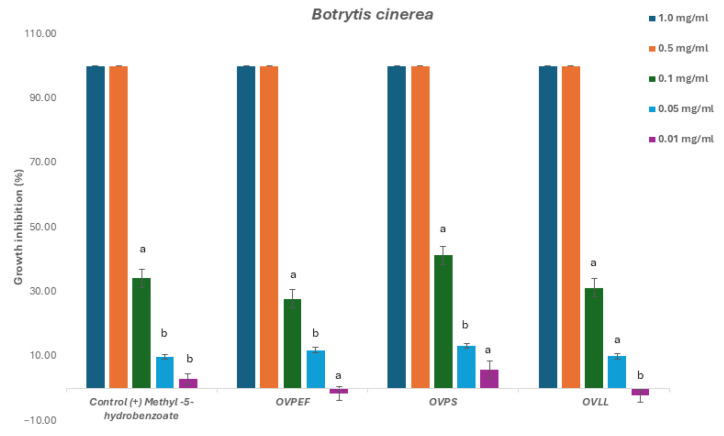
Antifungal effects (% growth inhibition) of the chemotypes of *O. vulgare* subsp. *virens* EOs against *Botrytis cinerea.* ^a,b^ Statistically significant differences by ANOVA, Tukey’s test, *p* < 0.05.

Results obtained for *Alternaria alternata* revealed slightly higher activity for the thymol chemotype OVPS. For this phytopathogenic fungus, all tested EOs and the control methyl-4-hydrobenzoate were inactive at the lowest concentration of 0.01 mg/mL (Figure 2). Likewise, antifungal activity for *Botrytis cinerea* led to a similar effect, with the thymol chemotype OVPS showing the lowest IC_50_ value of the other chemotypes in this study. All the EOs showed low activity for concentrations below 0.05 mg/mL (Figure 3).

**Figure 4:**
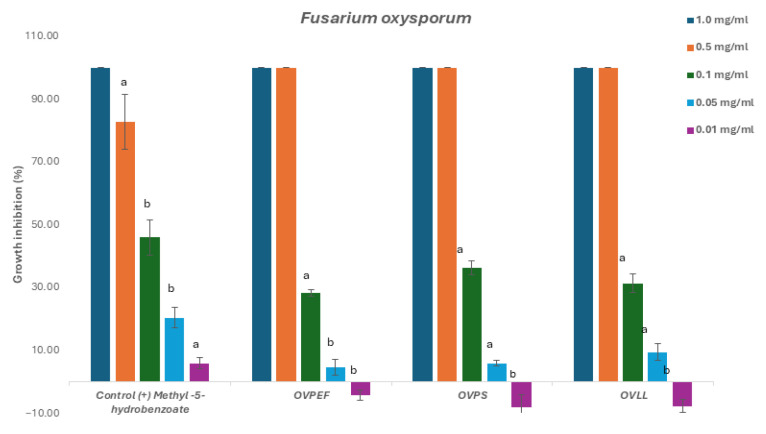
Antifungal effects (% growth inhibition) of the chemotypes of *O. vulgare* subsp. *virens* EOs against *Fusarium oxysporum.* ^a,b^ Statistically significant differences by ANOVA, Tukey’s test, *p* < 0.05.

*Fusarium oxysporum* showed similar behavior; OVPS EO had the lowest IC_50_ value, whereas OVPEF had a higher value, and the same for OVLL. As observed for *Botrytis cinerea*, all EOs were inactive at 0.01 mg/mL (Figure 4).

The results support the hypothesis that the fungicidal effect of each chemotype is influenced by the specific toxicity of their main active constituents or synergic interactions among multiple components, as observed by Zinno et al. [22]. The greater potency of thymol-rich oils is consistent with reports that thymol’s hydroxyl group enhances its ability to disrupt fungal membranes and interfere with ergosterol biosynthesis [38,39] Adebayo et al. [40] reported that a carvacrol chemotype *O. vulgare* subsp. *virens* EO inhibit the mycelial growth of *Botrytis cinerea* in a dose-dependent manner, significantly reducing growth at a concentration of 0.051 mg/mL at 24 h. Hou et al. [38] describe similar activity for *O. vulgare* subsp. *virens* on *Botrytis cinerea*, observing a complete inhibition for mycelial antifungal activity at 0.5 mg/mL and IC_50_ values of 0.140 mg/mL, whereas IC_50_ values for carvacrol and thymol were 0.009 and 0.021 mg/mL, respectively. Zhang et al. [41] confirmed the antifungal properties of thymol and carvacrol against *Botrytis cinerea*, reporting a minimum inhibitory concentration (MIC) and minimum fungicidal concentration of 0.065 mg/mL and 0.10 mg/mL for thymol, and 120 µL/L and 140 µL/L for carvacrol.

These results show variable outcomes: while some authors found carvacrol more effective than thymol against *Botrytis cinerea*, others reported the opposite. Such discrepancies may reflect strain-specific resistance or differences in oil composition beyond the major phenolics. Our results support the view that chemotype strongly influences antifungal performance, but that both thymol and carvacrol contribute significantly, with possible synergistic or antagonistic interactions involving minor constituents.

The higher antifungal activity of the thymol chemotype towards *Alternaria alternata* observed in the present study is in accordance with Perina et al. [42], who evaluated *Thymus vulgaris* EO and its major compound thymol, and reported MIC values of 0.50 and 0.25 mg/mL, respectively, results that were notably better than the MIC of 1.25 mg/mL observed for a commercial fungicide under the same conditions. Similarly, the observed efficacy against *F. oxysporum* is consistent with Bounar et al. [43], who reported MICs within the range 0.072–0.145 mg/mL, reinforcing the relevance of thymol and carvacrol as key antifungal agents in *O. vulgare* EOs.

#### 2.2.2. Obligate Biotrophic Fungi

The antifungal activity of *O. vulgare* subsp. *virens* chemotypes against *Oidium farinosum* was assessed using an in vivo conidial germination assay, which evaluates the ability of spores to germinate and initiate hyphal growth on leaves. This assay allows a direct comparison of EO efficacy at the earliest stages of fungal colonization, providing insight into their potential as preventive biocontrol agents. Table 3 summarizes the measured mycelial area as a proxy for conidial germination and early hyphal growth, reinforcing the relative potency of each chemotype in preventing fungal establishment.

The thymol- and carvacrol-rich chemotypes strongly suppressed conidial germination, producing reductions in early hyphal development that exceeded those of the commercial biopesticide ARAW™. In contrast, the mixed thymol–carvacrol chemotype OVLL was slightly less effective, suggesting that the relative abundance of individual monoterpenoids may influence activity. One possible explanation is a dilution effect by inactive constituents such as γ-terpinene and p-cymene, which may reduce the effective concentration of active compounds at the site of spore germination. This finding highlights the importance of EO chemical composition, rather than total monoterpenoid content alone, in determining antifungal potency.

These observations align partially with those of previous studies. Mirahmadi and Shayganfar [44] reported strong inhibitory effects of thymol–carvacrol chemotype EO from *Thymus daenensis* against three *Aspergillus* spp. fungi species, supporting the role of these phenolic monoterpenes in antifungal activity. Lambert et al. [45] proposed through mathematical modelling that the antifungal activity of *O. vulgare* EOs could be predicted based on specific formulations of carvacrol and thymol, attributing the total inhibitory effect to the additive action of each compound. Our data, however, suggest that in vivo efficacy is not strictly additive, as the EO with the highest combined thymol + carvacrol content did not outperform those with lower totals. This underscores the potential influence of synergistic or antagonistic interactions among minor EO constituents in shaping biological activity.

Furthermore, the requirement of higher concentrations (>10 µL/mL) to achieve effective inhibition indicates a threshold effect for antifungal activity, which may be relevant for practical application in crop protection. Overall, these results suggest that the monoterpenoids thymol and carvacrol are the principal drivers of spore inhibition, but that EO composition and minor constituents modulate the outcome in a manner that cannot be predicted solely from major component content.

### 2.3. Antifeeding Bioassay

The antifeeding potential of *O. vulgare* subsp. *virens* chemotype EOs was evaluated on fifth instar larvae of *Chrysodeixis chalcites* using a choice (election) and no-choice (non-election) leaf-disk bioassay. In this system, a refusal rate (FR) > 50% is considered indicative of significant feeding inhibition, with high FR values in both assays reflecting a strong deterrent effect [46,47]. Samples assessed with a combination of FR < 50% for both assays are considered inactive.

Statistical analysis was performed using the Wilcoxon signed-rank test to compare FR values between treatments within each assay. Additionally, differences between chemotypes were assessed with a Kruskal–Wallis test followed by Dunn’s post hoc test (*p* < 0.05) to identify which chemotypes significantly differed in their deterrent effects (Table 4).

The results revealed a distinct pattern of feeding deterrence among the chemotypes. Both the thymol-rich (OVPS) and carvacrol-rich (OVPEF) EOs induced substantial avoidance in the non-election assay (FR = 73.9% and 65.9%, respectively), indicating that larvae significantly reduced consumption when no alternative food was available. However, FR values in the election assay were below 50% (OVPS: 25.5%; OVPEF: 36.4%), suggesting that when given a choice, larvae still fed on treated disks, albeit less than untreated controls. Statistical comparisons confirmed that OVPS was significantly more effective than the mixed chemotype OVLL (FR = 68.71%) in the non-choice assay (*p* < 0.05), implying that equal proportions of these monoterpenoids may moderate the antifeeding effect, possibly due to minor constituents influencing palatability.

These findings indicate that thymol and carvacrol are the principal drivers of feeding deterrence in *C. chalcites*, consistent with previous reports on monoterpenoid activity against *Lepidopteran* larvae [48,49]. The higher FR observed in non-choice assays underscores the potential of these EOs as preventive biopesticides, as larvae are likely to avoid treated foliage in the absence of alternatives.

The mechanism of action of essential oils as insecticides is not clear. However, antifeedant, repellent, and toxic activities have been observed, demonstrating EOs’ potential to affect the physiology of insects in different ways, including by molting and respiratory inhibition, growth and fecundity reduction, and cuticle disruption [19]. Pavela [50] evaluated the efficacy of 30 aromatic compounds and their mutual binary combinations for acute toxicity against the larvae of *Spodoptera littoralis*, concluding that thymol, carvacrol, and trans-anethole were the most effective. In another study by Park et al. [51], EO extracted from *Thymus vulgaris* showed strong mosquito repellent activity attributed to its high carvacrol content.

Overall, statistical analysis supports that thymol- and carvacrol-rich EOs significantly deter feeding, whereas the mixed chemotype exhibits intermediate effects, likely due to partial dilution of the active monoterpenoids. These results reinforce the importance of chemotype-specific selection when developing EO-based biopesticides for lepidopteran pests.

### 2.4. Mortality and Oviposition Bioassay

The effects of two *O. vulgare* subsp. *virens* chemotype EOs (thymol-rich OVPS and carvacrol-rich OVPEF) on adult mortality and oviposition of *Ceratitis capitata* were assessed using a no-choice bioassay with artificial fruits. Mortality was evaluated at 24, 48, and 72 h and corrected using Abbott’s formula, while oviposition deterrence was quantified as% OD. Statistical comparisons were performed using the Wilcoxon signed-rank test (Table 5).

Overall, adult mortality induced by all EOs and pure compounds was low, not exceeding 16.2% even at 50 mg/mL. Even the positive control azadirachtin, tested at 0.88 mg/mL, resulted in very low adult mortality (4.7%), highlighting the inherent tolerance of adult *C. capitata* to both botanical and conventional insecticidal agents under these conditions.

Among the EOs, OVPEF (carvacrol-rich) showed the highest mortality (8.1%), while OVPS (thymol-rich) induced 6.8% mortality. This could be due to the presence of carvacrol as a major component, as described in the previous section concerning the phytochemical profile. This conclusion is further evidenced by the mortality rate observed for the standard monoterpenoid carvacrol (16.2%), which was significantly higher than that of thymol (14.9%, *p* < 0.05). Indeed, this compound has been recognized as toxic and repellent for other arthropods, including *Spodoptera littoralis* larvae, with lethal dose LD_50_ values < 0.100 mg per larvae [48,50] and for mosquito *Culex pipiens pallens* [51]. Karpouhtsis et al. [52] reported carvacrol to be more toxic than thymol for second instar larvae of the fruit flies *Drosophila melanogaster*. Other minor EO constituents, including carvacrol methyl ether and p-cymene, had negligible effects, confirming that major phenolics primarily drive EO bioactivity. The study by Pavela [50] on the effect of a combination of binary mixture carvacrol-thymol, applied in doses equivalent to lethal dose LD_25_, showed a larval mortality of 32.4% and a synergistic effect with potential in the creation of new formulation for botanical insecticides. These findings suggest that although adult flies are relatively insensitive to acute toxicity, the phenolic compounds in *O. vulgare* subsp. *virens* EOs still confer measurable toxic effects, consistent with previous observations.

As for the oviposition deterrent rate, all chemotype samples were found to be ineffective in preventing egg laying by *C. capitata*, with negative oviposition deterrent rate (OD) values for OVPEF (−177.8%) and OVPS (−94.1%), indicating stimulation rather than inhibition of egg laying. Statistical analysis confirmed that these values were significantly different from those of the positive control azadirachtin (49.3%, *p* < 0.05). This ineffective behavior was also observed for the main components, with standard carvacrol and thymol inducing negative oviposition rates (−172.4% and −48.1%, respectively). This stimulation in the oviposition of *C. capitata* on the surface of artificial fruits is puzzling, since it contrasts with the Sedy et al. study on other insect species, such as *Frankliniella occidentalis*, where carvacrol significantly reduced oviposition [53], highlighting species-specific responses. However, although thymol and carvacrol are oviposition deterrents in several insect pests, there is evidence that both compounds can act as short-range attractants *for C. capitata*, at least for males of this species [54]. It is possible that this effect is responsible for the oviposition stimulation detected in the present work for thymol and carvacrol-treated artificial fruits, and concomitantly with this result, also for *O. vulgare* subsp. *virens* EOs from the two thymol and carvacrol-rich chemotypes.

Interestingly, minor compounds such as carvacrol methyl ether displayed positive deterrent effects, although these were masked within the full EO due to the predominance of thymol and carvacrol. α-Pinene and p-cymene, despite low acute toxicity, demonstrated moderate oviposition deterrence (59.3% and 28.5%, respectively),

These findings indicate that *O. vulgare* subsp. *virens* EOs exert limited direct activity in adults but may modulate behavioral responses, particularly oviposition. The differential effects of major and minor EO constituents underscore the importance of chemical composition in designing formulations for behavioral manipulation, such as baited traps or oviposition-deterrent coatings.

### 2.5. In Vitro Inhibition Assays

The in vitro inhibition assays were conducted to elucidate the mechanisms of action of the EOs of the different *O. vulgare* subsp. *virens* chemotypes, as well as their main compounds—carvacrol and thymol. The assays aimed to evaluate whether the in vivo effects were due to action on the nervous or gastrointestinal system.

#### 2.5.1. Acetylcholinesterase Inhibitory Assay

Carvacrol, thymol, and the three chemotypes of *O. vulgare* subsp. *virens* were evaluated for their ability to inhibit AChE. A carvacrol: thymol solution (1:1) was prepared to mimic the chemotype OVLL. The results are summarized in Table 6.

The EOs and pure compounds significantly inhibited AChE in a dose-dependent manner, with IC_50_ values ranging from 0.027 ± 0.001 mg/mL for carvacrol to 0.135 ± 0.003 mg/mL for the thymol chemotype EO (OVPS). Statistical analysis (ANOVA followed by Tukey’s post hoc test) confirmed that carvacrol exhibited significantly stronger AChE inhibition than thymol (*p* < 0.05), while the EOs showed intermediate activity, reflecting the relative abundance of phenolic monoterpenes. The IC_50_ of the 1:1 carvacrol: thymol mixture (0.070 ± 0.002 mg/mL) closely matched the OVLL mixed chemotype (0.109 ± 0.015 mg/mL), suggesting additive or partially synergistic effects between the two compounds.

These findings align with previous studies showing that carvacrol is a potent AChE inhibitor in invertebrates, a behavior described previously by Jukic et al. [25] and Askin et al. [23], although the observed lack of correlation between AChE inhibition and adult *C. capitata* mortality indicates that other physiological or behavioral factors may modulate susceptibility. Notably, γ-terpinene, a compound present in OVPS and OVLL EOs and previously reported as an AChE inhibitor [55], did not show significant AChE inhibition under our experimental conditions suggesting that minor EO constituents contribute less to their overall neurotoxic effects.

Invertebrate AChE activity can be decreased by plant components, raising the possibility of using them in the formulation of insecticides. The most active compounds are usually alkaloids, such as galantamine, used in the study as positive control. Thus, our data support the hypothesis that AChE inhibition by EOs may contribute to the reduced larval feeding or behavioral changes observed, rather than acute adult mortality.

#### 2.5.2. α- and β-Glucosidase Inhibitory Assays

Inhibition of digestive enzymes is an important component of plant defense, as these inhibitors target insect gut hydrolases that are essential for nutrient utilization. Many insect pests rely on α-amylase and α-glucosidase (α-GLU) to break down carbohydrates for energy. α-GLUs are present in the alimentary canal, salivary secretions, and hemolymph. β-Glucosidase (β-GLU) is a key enzyme in herbivorous insects, enabling digestion of plant cell walls and detoxification of plant metabolites. In plants, β-GLU hydrolyzes glycosides, activating protoxins that serve as a defense against herbivores. Conversely, insect-derived β-GLU can remove sugar moieties from these compounds, deactivating protoxins, which increases the palatability of plant tissues and reduces their toxicity [56]. In the present study, only β-GLU from plant origin (almond) was evaluated, thus evaluating whether thymol and carvacrol help or impair the plant defense against herbivore insects.

Even though this study evaluated the in vivo effects of *O. vulgare* subsp. *virens* chemotypes and their main components on two insects, it was evident at some point that *C. chalcites* is not a worrying pest for local agriculture, being very difficult to find in the field, so *C. capitata* became our main interest to proceed to in vitro assays. Since *C. capitata* feeds on fruit, a low inhibition of plant β-GLU is considered a positive outcome, while a good inhibition of α-GLU is of interest.

Carvacrol, thymol, and the EO from the three chemotypes of *O. vulgare* subsp. *virens* were evaluated for their ability to inhibit digestive enzymes, namely α- and β-GLU. The results are summarized in Table 7.

The EOs and standards displayed markedly distinct inhibitory profiles against α-GLU. Thymol standard was the most potent α-GLU inhibitor (IC_50_ = 0.304 ± 0.025 mg/mL), followed by the 1:1 carvacrol: thymol mixture (0.497 ± 0.091 mg/mL), while carvacrol standard and the carvacrol-rich EO (OVPEF) exhibited minimal activity. Among the chemotypes, OVPS (thymol-rich) showed moderate α-GLU inhibition (IC_50_ = 16.33 ± 8.15 mg/mL), whereas OVLL (mixed) and OVPEF (carvacrol-rich) were largely inactive. Statistical comparisons (one-way ANOVA, Tukey’s test, *p* < 0.05) confirmed that OVPS significantly inhibited α-GLU compared to OVPEF and OVLL.

As to the β-GLU inhibitory assay, carvacrol and thymol presented high calculated IC_50_ values. Therefore, the EOs of the three oregano chemotypes also exhibited high IC_50_ values. Thus, neither the pure compounds nor the EOs inhibited plant β-GLU.

The selective α-GLU inhibition by thymol aligns with its role in deterring herbivore feeding by reducing carbohydrate digestibility. In contrast, β-GLU, representing plant or herbivore-derived glucosidases relevant for detoxification, was largely unaffected by all EOs and standards, suggesting that the oils do not compromise plant defensive metabolism, a desirable trait for minimizing negative impacts on host plants.

Overall, the enzyme inhibition data provide insight into the antifeeding activity observed in *C. chalcites* bioassays. The correlation between thymol content and α-GLU inhibition supports the conclusion that thymol mediates reduced larval feeding, while carvacrol primarily contributes to neuroactive effects via AChE inhibition. These results are consistent with previous reports demonstrating the dual-mode action of monoterpenes carvacrol [57], thymol [58], and *O. vulgare* subsp. *virens* EO [59,60] in insect deterrence, combining digestive enzyme interference and neurotoxicity; however, few have explored their mechanisms of action on enzymes relevant to insect development.

## 3. Materials and Methods

### 3.1. Chemicals and Standards

Standards used for identification purposes with GC-FID chromatography were as follows: ƴ-terpinene (99%), α-terpinene (95%), p-cymene (99%), (-)-α-pinene (98%), and (-)-β-pinene (99%) were acquired from Fluka™ (Buchs, Switzerland). Thymol (99%), carvacrol (98%), and carvacrol methyl ether (98%) were acquired from Merck KGaA™ (Darmstadt, Germany). *n*-Hexane (95%) was acquired from PanReac AppliChem™ (Barcelona, Spain).

For testing fungicide/fungistatic activity, potato glucose agar (PGA), and tetracyclin were acquired from Sigma-Aldrich™ (St. Louis, MO, USA), while phytofungicide methylparaben (methyl-4-hydrobenzoate) was acquired from Acros™ (Geel, Belgium), and a formulation similar to the phytofungicide ARAW™ was prepared in our laboratory using the manufacturer’s technical sheet (3.2% p/p eugenol; 6.4% p/p geraniol; 6.4% p/p thymol) (SIPCAM PORTUGAL, Vila Nova da Rainha, Portugal). For the antifeeding assay, agar was acquired from Sigma-Aldrich™ (St. Louis, MO, USA) and for the insecticidal assay, yeast hydrolysate was acquired from Fluka™ (Buchs, Switzerland). For the in vitro inhibitory assay, AChE from Electrophorus electricus was acquired from Sigma-Aldrich™ (St. Louis, MO, USA), 5,5′-dithiobis (2-nitrobenzoic acid) 99% (DTNB, Ellman’s Reagent) was acquired from Thermo Fisher Scientific™ (Waltham, MA, USA), and acetylthiocholine iodide (ATChI) was acquired from TCI™ (Tokyo, Japan). AChE inhibitor galanthamine hydrobromide was sourced from Merck KGaA (Darmstadt, Germany). The digestive enzymes α-glucosidase (α-GLU) extracted from *Saccharomyces cerevisiae* and β-glucosidase (β-GLU) from almonds were acquired from Sigma-Aldrich™ (St. Louis, MO, USA), as well as their substrates 4-nitrophenyl α-D-glucopyranoside (α-pNPG) and 4-nitrophenyl β-D-glucopyranoside (β-pNPG). The common inhibitor of those enzymes, 1-deoxynojirimycin (1-DNJ), was acquired from Chengdu Biopurify Phytochemicals Ltd. (Chengdu, China).

### 3.2. Sample Preparation

For this study, samples from *Origanum vulgare* subsp. *virens* (Hoffmanns. & Link) Bonnier & Layens were selected. Three samples were evaluated: *O. vulgare* subsp. *virens* from Ponta do Sol—Madeira (OVPS) and *O. vulgare* subsp. *virens* from San Cristóbal de La Laguna—Tenerife, Canary Islands (OVLL) were obtained in local markets as dried aerial parts; *O. vulgare* subsp. *virens* from Parque Ecológico do Funchal—Madeira (OVPEF) was collected in the wild and dried in the laboratory. Samples for OVPS, OVLL, and OVPEF have been deposit in the Madeira Botanical Garden *Eng. Rui Vieira* and voucher numbers have been added accordingly. (OVPS—MADJ306224; OVLL—MADJ306225; OVPEF—MADJ306206). All three samples were identified by Dr. Fátima Rocha, botanist at the Agricultural Quality Laboratory, Directorate of Agricultural and Agri-Food Laboratories Services.

Leaves were dried for 48 h, evaluated for humidity content using a Kern DBS humidity scale (Kern-Sohn, Ballinger, Germany) and ground to fine powder in a mechanical grinder until achieving particle sizes < 250 µm.

### 3.3. Essential Oil Extraction

The EOs were obtained by hydrodistillation for 4 h at a 1:20 ratio dry plant: water (*w*/*v*) using a Clevenger-type apparatus. Following distillation, the oil phase was separated, collected, and filtered to remove residual water. The oils were then dried with anhydrous sodium sulfate to eliminate traces of moisture and further dried under a gentle stream of nitrogen. Finally, the oils were stored in amber vials at 4 °C until further analysis.

### 3.4. Phytochemical Analysis

#### 3.4.1. Gas Chromatography–Flame Ionization Detector (GC–FID) Conditions

The samples were prepared by adding 1 mL of n-hexane to a portion (20 mg) of each EO and analyzed using a gas chromatography with flame ionization detector (GC–FID) method. Different standards were also analyzed, using a gradient concentration calibration curve (0.25–10 mg/mL) for quantification.

The EO characterization was performed using an Agilent 7890A gas chromatography (Agilent, Santa Clara, CA, USA) equipped with an autosampler Agilent 7693. The column used was a SPB™FA fused silica capillary column (30 m × 0.25 nm) with 0.20 µm film thickness (Supelco, Bellefonte, CA, USA) (Supplementary Materials). Data analysis was carried out using Agilent ChemStation proprietary software A.01.04 and statistical analysis was evaluated by one-way analysis of variance (ANOVA) followed by Tukey’s multiple comparison test using IBM SPSS Statistic 29.0.1.0 software (IBM Corp, Armonk, NY, USA). Principal component analysis was performed by MetaboAnalyst 6.0 software (Alberta, CA, USA).

Method validation was evaluated based on linearity and sensitivity, including the limit of detection (LOD) and limit of quantification (LOQ). Linearity was assessed by varying the standard concentrations and plotting the relative area against concentration. The LOD and LOQ were calculated by multiplying the standard deviation of the calibration curve by three and ten times, respectively, by the regression slope.

#### 3.4.2. Gas Chromatography–Mass Spectrometry (GC–MS) Conditions

A 10 mg portion of each essential oil (EO) was dissolved in methanol at a 1:50 ratio, then filtered and injected into the GC–MS. The separation and qualitative analysis were carried out using an Agilent 6890N gas chromatograph (Agilent, Santa Clara, CA, USA) equipped with an Agilent 5975 Inert Mass Selective Detector. An Agilent J&W HP-5 column, a nonpolar (5%-phenyl)-methyl polysiloxane column (300 × 0.32 mm I.D.) with a 0.25 µm film, was used. Chromatographic peak identification was conducted using the NIST Mass Spectral Search Program v2.2—2005 software (National Institute of Standards and Technology, Gaithersburg, MD, USA). To obtain reference retention indices for the identified compounds and compare them with literature values under similar experimental conditions, the Supelco C7–C40 Saturated Alkanes Mix Standard (St. Louis, MO, USA) was analyzed under identical conditions.

### 3.5. Fungicide and Fungistatic Activity

#### 3.5.1. Phytopathogenic Fungi

The colonies of *Fusarium oxysporum*, *Alternaria alternata*, and *Botrytis cinerea* were obtained from Colección Española de Cultivos Tipo (CECT) and maintained on potato glucose agar (Sigma-Aldrich™, Steinheim, Germany) at 25 ± 1 °C under obscurity at Laboratório de Fitopatología de la Universidad de La Laguna. To obtain a large population of pure colonies, a seven-day colony-repicking schedule to fresh medium was established.

The agar dilution methodology devised by Griffin et al. [61], with some modification, was selected for the evaluation of in vitro antifungal activity [62]. For each EO, a stock solution of 50 mg/mL in ethanol 96% was prepared. For culture medium, potato glucose agar was prepared at a concentration of 39 g/L, with the addition of 10 µg/mL of tetracycline (Sigma-Aldrich™, Steinheim, Germany), a broad-spectrum antibiotic. The stock solution was merged into 4.9 mL aliquots of molten culture medium at five different gradient concentrations: 0.01, 0.05, 0.1, 0.5, and 1.0 mg/mL and poured into sterile Petri dishes (90 mm diameter). Blank culture medium Petri dishes were prepared for negative control and phytofungicide methylparaben was used for positive control. A disk of inoculum, approximately 6 mm in diameter, was removed with a special tool from 7-day-old cultures and deposit in the PGA dishes corresponding to different concentration, eight per dish, at equidistant points. The dishes were incubated at 25 ± 20 C, under darkness. The incubation time was 48 h for *Botrytis cinerea* and 72 h for *Fusarium oxysporum* and *Alternaria alternata*. The mycelium growth or inhibition was monitored by digitalizing the photos of the dishes and comparing the diameters of the treated colonies with the control ones, using the image processing software Image J 1.43 (Bethesda, MD, USA). The percentage of mycelia inhibition was analyzed by ANOVA variance using IBM SPSS Statistic 29.0.1.0 software (IBM Corp, Armonk, NY, USA), and the half-maximal inhibitory concentration (IC_50_) was estimated by simple linear regression using GraphPad Prism 10 (GraphPad Software, Boston, MA, USA).

#### 3.5.2. Obligate Biotrophic Fungi

The evaluation of antifungal activities for biotrophic fungi associated with the powdery mildew disease was assessed by an in vivo moisture chamber isolation methodology. This method evaluates the ability of spores to germinate and initiate hyphal growth on leaves, being referenced as a conidial germination assay [63]. Young leaves from an orchard apple, *Malus domestica*, infected with *Oidium farinosum* were collected and placed in a Petri plate with enough water to maintain a humidity content between 80 and 100%. For each EO, 400 µL of six different gradient concentrations in ethanol 96% (2.5, 5.0, 7.5, 10.0, 15.0, and 20.0 µL/L) and ethanol 0.5% + Tween 20 1% (10, 50, 100, 200, 500, and 2000 µg/mL were evaluated. The solvent was used for negative control and commercial fungicide Methylparaben™ and a homemade phytofungicide ARAW™ were selected as positive controls. The assay lasted up to 14 days. Conidial germination and early hyphal development were monitored at incubation, 8 days, and 14 days by measuring the mycelial area on the leaf surface.

### 3.6. Insect Bioassay

#### 3.6.1. Breeding and Maintenance of Insects

Breeding and maintenance of larvae of *Chrysodeixis chalcites* were performed in a moisture chamber at 24 ± 1 °C, with relative humidity in the range 60–70% and a 16:8 h photoperiod (light: obscurity). Feeding consisted in a semi-synthetic diet described by Bajonero and Parra [64], and the colony was maintained for more than 10 generations.

For *Ceratitis capitata*, adult specimens were obtained from a colony maintained for more than 20 generations. Laboratory conditions were the following: 14:10 h photoperiod (light: obscurity), temperature of 24 ± 1 °C, and humidity of 70 ± 5%. An artificial diet, according to the methodology described by Quesada-Moraga et al. [65], was used for larvae feeding and adults were supplemented with a mixture of water and hydrolysate yeast and sugar (1:3 *w*/*w*).

#### 3.6.2. Antifeeding Bioassay

The experimental setting was based on the design described by Escoubas et al. [66] and González-Coloma et al. [46], based on a leaf-disk election/non-election bioassay. Disks of 9 cm diameter were cut out from banana (*Musa acuminata*) leaves to function as substrate, inserted in Petri dishes, and covered with agar 3% (Sigma-Aldrich™, Steinheim, Germany) disks, for support, one per dish. With a special tool, nine holes/wells were prepared on each Petri dish, creating a contact surface for the deposition of extract or control in the banana disk. EOs solutions were prepared at concentration of 40 mg/mL in ethanol 96%. Election assays are arranged for each dish with five wells treated with 5 µL of EO stock solution and four controls with ethanol 96%, alternating for the remaining replicates, for a total of six plates and 0.2 mg/cm2 of area per well. For the no-election assay, each dish corresponded to either treatment or control. The pathogen corresponded to the five instar larvae stage of *Chrysodeixis chalcites*, three specimens for each dish. The assay ended 8–10 h after larval addition or when 50% consumption of the control areas was observed. The consumed area for each dish was monitored using image processing software Image J 1.43 (Bethesda, MD, USA) and expressed in terms of insect antifeeding rate or refusal rate (FR) (Supplementary Materials Equation (S1)).

The refusal rate was analyzed according to non-parametric Wilcoxon signed-rank test using IBM SPSS Statistic 29.0.1.0 software (IBM Corp, Armonk, NY, USA).

#### 3.6.3. Mortality and Oviposition Bioassay

Each EO was evaluated for its effect on mortality and oviposition for the fruit fly *Ceratitis capitata* using the no-choice test with artificial fruits, according to a methodology adapted and improved based on the work of Salles [67], Furtado et al. [68], and Tavares et al. [14]. The artificial fruits were produced from a solution containing 350 mL of distilled water, 75 mL of natural orange juice, 8.0 g of agar, and 4 mL of methyl hydroxybenzoate solution 10% in ethanol, which was poured into half-sphere forms (v = 11 mL, each). The spherical artificial fruits were wrapped in parafilm and placed in plastic cups (8 cm of nozzle diameter by 10.5 cm of height) where they were brushed with 150 µL of 10% honey solution and left to dry for 1 h. Then, 250 µL of the different essential oils and standards at a concentration of 50 mg/mL in ethanol 96% were applied and brushed evenly over the surface of the fruit. Azadirachtin (Sigma-Aldrich™, St. Louis, MO, USA) was included as a positive control because it is a well-established botanical insecticide with documented insecticidal, antifeedant, and oviposition deterrent effects across diverse insect taxa. Although azadirachtin is generally more effective against immature stages than adults, it has also been reported to reduce oviposition in *Diptera* flies. In this study, azadirachtin was applied at a concentration of 0.88 mg/mL (0.08% w:v), which lies within the commonly used range (0.1–5 mg/mL) for ingestion and contact assays, ensuring its suitability as a benchmark for comparison with the essential EOs tested. After a drying period of 2–4 h, 13 flies (3 males and 10 females) were selected and added to the cup. Mortality was checked every 24 h, with a maximum duration of 72 h. After that period, the artificial fruits were removed from the cups for a verification of the oviposition. Each experiment was replicated 12 times. The percentage mortality was corrected by Abbott’s formula when the control mortality > 5%. The numbers of eggs laid on treated and control groups of fruits were used for the determination of oviposition deterrent activity (Supplementary Materials Equation (S2)).

Mortality and oviposition index were calculated by non-parametric Kolmogorov–Smirnov test using IBM SPSS Statistic 29.0.1.0 software (IBM Corp, Armonk, NY, USA).

### 3.7. In Vitro Inhibition Assays

#### 3.7.1. Acetylcholinesterase Inhibition Assay

The activity of AChE was assessed using ATChI as a substrate. AChE catalyzes the hydrolysis of ATChI, producing thiocholine, which subsequently reacts with Ellman’s reagent. This reaction generates 5-thio-2-nitrobenzoic acid, which was quantified by measuring its absorbance at 405 nm. The assay was performed as described by Barreto and Simões [69], with slight modifications. Stock solutions of EOs and standards were prepared at concentrations of 50 mg/mL and 25 mg/mL in ethanol, respectively. To prevent any solvent-related inhibition of the enzyme, 12 μL of these stock solutions were diluted with 1988 μL of phosphate buffer. In a 96-well microplate, 240 μL of EO/standard solution was pipetted into the wells in column 2. In the remaining wells (columns 3–12), 120 μL of phosphate buffer was added. Using a multichannel micropipette, 120 μL was transferred from wells in column 2 to wells in column 3. After homogenization, the procedure was repeated until column 11 (Supplementary Materials Figure S2). Then, 110 μL of phosphate buffer and 20 μL of 0.25 U/mL AChE solution were added to all wells from column 2 to 12. After a period of incubation (5 min), 20 μL of substrate mixture (equal parts of 3 mM DTNB and 75 mM ATChI) was added and the absorbance was read at 405 nm (Multiskan FC, Thermo Fisher Scientific™, MA, USA) at 0 s, 150 s, 300 s, and 450 s. The control represented 100% enzyme activity, so EO or standard was replaced by buffer. Each assay was conducted in duplicate. Reaction rates (v, ∆Abs _405 nm_/min) were determined by measuring the variation in absorbance over time for each well and the percentage inhibition was calculated (Supplementary Materials Equation (S3)).

The inhibitory activity was expressed as the IC_50_ value (mg/mL), determined from the least-squares regression of the logarithmic concentrations plotted against the percentage inhibition. This value reflects the concentration of EO or standard needed to achieve a 50% reduction in enzyme activity compared to the control.

#### 3.7.2. α-GLU Inhibitory Assay

The inhibition of α-GLU activity was determined by measuring the amount of p-nitrophenyl obtained through the hydrolysis of α-pNPG by the enzyme. This assay was performed based on a previously established method [70] with some adaptations [71] Stock solutions of EO/standards were prepared at concentration of 100 mg/mL in DMSO. To prevent any solvent-related inhibition of the enzyme, 12 μL of these stock solutions was diluted with 1988 μL of phosphate buffer. In a 96-well plate, 50 µL of EO/standard solution (sequential dilution) and 50 μL of α-pNPG solution (5 mM) were mixed with 50 μL of α-GLU solution (0.01 mg/mL) (Supplementary Materials Figure S3). All solutions were prepared in 0.1 M phosphate buffer (pH 7.0). After 20 min incubation at 37 °C in the dark, 100 μL of sodium carbonate aqueous solution (0.1 M) was added and the absorbance was read at 405 nm (Multiskan FC, Thermo Fisher Scientific™, MA, USA). 1-DNJ was used as a positive control in the experiment. The α-GLU control (C) represented 100% enzyme activity, where the EO/standard was replaced with buffer. Sample blanks (SB) were included for each sample to correct background absorbance, replacing the substrate with buffer. A control blank (CB) was prepared using only the buffer (replacing both the EO/standard and substrate).

The inhibitory activity was expressed as the IC_50_ value (mg/mL), determined as the same as AChE inhibition assay.

#### 3.7.3. β-GLU Inhibitory Assay

The inhibition of β-GLU was assessed using the procedure outlined in Section 3.7.2., using β-pNPG as substrate and a β-GLU solution at concentration of 0.1 mg/mL (Figure S8—Supplementary Materials).

## 4. Conclusions

This study characterized the chemical composition and bioactivity of three *Origanum vulgare* subsp. *virens* (Hoffmanns. & Link) Bonnier & Layens chemotypes: thymol-rich (OVPS), carvacrol-rich (OVPEF), and a mixed thymol: carvacrol chemotype (OVLL). Major monoterpenoids—thymol and carvacrol—were identified as the principal active constituents, while minor compounds such as γ-terpinene, p-cymene, α-pinene, and β-caryophyllene contributed minimally to the observed biological effects.

All chemotypes demonstrated antifungal activity against the tested phytopathogenic fungi at concentrations above 0.5 mg/mL. In antifeeding bioassays, *Chrysodeixis chalcites* larvae exhibited high refusal rates in non-choice assays, indicating strong feeding deterrence, while choice assays showed moderate effects, highlighting the role of monoterpenoid concentration and chemotype in influencing palatability. Adult *Ceratitis capitata* were largely tolerant of the essential oils, with mortality not exceeding 16.2%, and oviposition was not inhibited; OVPEF displayed the highest adult toxicity (8.1%), likely attributable to its carvacrol content. Carvacrol exhibited the strongest AChE inhibition, whereas thymol primarily inhibited α-glucosidase, with no significant β-glucosidase inhibition observed.

These findings suggest that *O. vulgare* subsp. *virens* EOs, particularly OVPEF and OVPS, are most effective as behavioral modulators (feeding deterrents and oviposition influencers) and as biofungicides rather than as direct adult insecticides. Their chemotype-specific activity underscores the importance of selecting the appropriate EO composition for practical applications. Integrating these essential oils into environmentally safe and economically viable integrated pest management (IPM) strategies, either alone or in combination with other biological agents, could help control phytopathogenic fungi and deter herbivorous insect pests. Future work should focus on optimizing binary mixtures of thymol and carvacrol for synergistic effects and validating efficacy under field conditions.

## Figures and Tables

**Table 1:** Relative percentage (% ± standard deviation) of major constituents of *O. vulgare* subsp. *virens* EOs (*n* = *3*).

Classes of Compounds s	I.D.	RI ^1^	RI ^2^	OVPEF	OVPS	OVLL
				Relative% ± SD	Relative% ± SD	Relative% ± SD
Monoterpene	α-pinene	1011	910	0.40 ± 0.06 ^a^	1.61 ± 0.09 ^b^	2.11 ± 0.09 ^b^
	α-terpinene	1168	1120	0.77 ± 0.06 ^a^	n.d.	2.50 ± 0.12 ^b^
	γ-terpinene	1245	1043	5.97 ± 0.38 ^a^	14.81 ± 0.75 ^b^	18.77 ± 0.75 ^c^
	p-cymene	1272	1024	2.11 ± 0.29 ^ab^	3.57 ± 0.21 ^ab^	5.05 ± 0.30 ^b^
Methyl ether terpenes	Carvacrol methyl ether	1549	1231	1.96 ± 0.09 ^a^	0.87 ± 0.07 ^b^	4.71 ± 0.15 ^c^
Sesquiterpene	β-caryophyllene	1601	1439	0.78 ± 0.05 ^a^	1.12 ± 0.04 ^a^	0.54 ± 0,20 ^a^
Monoterpenoid	Thymol	2120	1288	5.65 ± 0.47 ^a^	59.19 ± 0.90 ^b^	30.22 ± 0.60 ^c^
	Carvacrol	2125	1290	73.04 ± 1.07 ^aa^	4.16 ± 0.35 ^b^	32.18 ± 1.54 ^c^
Other				9.32	14.68	3.93

Mean of three replicates; ^1^ linear retention indices relative to C7-C40 n-alkanes on a SPB PUFA polar column; ^2^ linear retention indices relative to C7-C40 n-alkanes on a HP-5 nonpolar column; n.d not detected; ^a–c^ indicates statistically significant differences among chemotypes according to one-way ANOVA, followed by Tukey’s test *p* < 0.05.

**Table 2:** Estimated IC_50_ (mg/mL) for *Alternaria alternata*, *Botrytis cinerea*, and *Fusarium oxysporum* after exposure to different chemotypes of *O. vulgare* subsp. *virens* EOs.

Code	Inhibition Concentration IC_50_ (mg/mL) and Gradient Concentration t = 48 ^1^ and 72 h					
	*Alternaria alternata*		*Botrytis cinerea*		*Fusarium oxysporum*	
	IC_50_ (mg/mL)	Pearson Correlation Coefficient r *p* Value	IC_50_ (mg/mL)	Pearson Correlation Coefficient r *p* Value	IC_50_ (mg/mL)	Pearson Correlation Coefficient r *p* Value
OVPEF (C)	0.121 (0.11–0.13)	0.9667 0.0073	0.138 (0.13–0.15)	0.9536 0.0119	0.131 (0.12–0.14)	0.9504 0.0132
OVPS (T)	0.109 (0.10–0.11)	0.9687 0.0066	0.113 (0.11–0.12)	0.9675 0.0070	0.118 (0.11–0.12)	0.9654 0.0077
OVLL (Mix)	0.126 (0.12- 0.13)	0.9480 0.0141	0.132 (0.12–0.14)	0.9570 0.0106	0.131 (0.12–0.14)	0.9659 0.0075
Control (+) methyl-4-hydrobenzoate	0.119 (0.11–0.13)	0.9613 0.009	0.192 (0.18–0.20)	0.9444 0.016	0.124 (0.11–0.14)	0.9873 0.002

IC_50_: Concentration of the extract that induces 50% of the mycelium inhibition (95% confidence interval, bottom and upper limits); ^1^ indicates incubation time for *Botrytis cinerea*.

**Table 3:** Effect of *O. vulgare* subsp. *virens* chemotype EO_S_ on *Oidium farinosum* conidial germination and early hyphal growth (% mycelial area per leaf).

Essential Oil	Mycelial Area per Leaf (%)					
	Gradient Concentration (µL/mL)					
	2.5 µL/mL	5 µL/mL	7.5 µL/mL	10 µL/mL	15 µL/mL	20 µL/mL
OVPEF (C)	83	79	82	37	28	21
OVPS (T)	85	80	83	49	44	26
OVLL (Mix)	86	81	84	52	42	35
Control (+) methyl-4-hydrobenzoate	31	26	28	22	28	18
Control (+) ARAW	41	42	38	33	31	32
Control (−) EtOH 96%	81	86	88	83	71	62

**Table 4:** Refusal rate (% FR ± standard deviation) for fifth instar *Chrysodeixis chalcites* larvae exposed to *O. vulgare* subsp. *virens* chemotype EOs at 50 µg/cm^2^, after 10 h.

Code	Refusal Rate (% FR ^1^) 50 µg/cm^2^, t = 10 h	
	*Chrysodeixis chalcites*	
	Election Assay	Non-Election Assay
OVPEF (C)	36.42 ± 1.87 ^b^	65.93 ± 2.97 ^a^
OVPS (T)	25.54 ± 2.05 ^c^	73.85 ± 8.46 ^a^
OVLL (Mix)	31.89 ± 2.04 ^bc^	68.71 ± 4.68 ^a^

^1^ % FR = [1-(T/C)] x 100 where T and C represent the% of area ingested for treated disk and control disk, respectively; ^a–c^ statistically significant differences within each assay (Kruskal–Wallis test with Dunn’s post hoc, *p* < 0.05).

**Table 5:** Adult mortality rate (% ± standard deviation) and oviposition deterrent rate (% OD ± standard deviation) for *Ceratitis capitata*.

Code	Concentration (mg/mL)	Mortality Rate (% ^1^)			Oviposition Deterrent Rate (% OD ^2^)
		Abbott 24 h	Abbott 48 h	Abbott 72 h	
OVPEF (C)	50	−0.64 ± 0.00 ^a^	1.34 ± 2.65 ^a^	8.11 ± 4.27 ^a^	−177.78 ± 83.87 ^b^
OVPS (T)		0.65 ± 0.87 ^a^	4.03 ± 3.36 ^a^	6.75 ± 3.80 ^a^	−94.09 ± 40.53 ^a^
α-pinene		0.65 ± 1.29 ^a^	3.36 ± 3.97 ^a^	7.43 ± 5.13 ^a^	59.34 ± 8.02 ^d^
Carvacrol		5.16 ± 3.03 ^a^	14.10 ± 6.45 ^a^	16.21 ± 6.72 ^a^	−172.44 ± 78.52 ^ab^
Carvacrol methyl ether		0.65 ± 1.29 ^a^	7.39 ± 6.23 ^a^	10.13 ± 6.65 ^a^	20.17 ± 21.41 ^abcd^
p-cymene		−0.64 ± 0.00 ^a^	−1.34 ± 2.09 ^a^	−0.00 ± 2.51 ^a^	28.48 ± 14.28 ^bc^
Thymol		2.58 ± 1.49 ^a^	11.41 ± 3.97 ^b^	14.86 ± 3.67 ^a^	−48.09 ± 35.22 ^abc^
Control (-) H_2_O		0.00 ± 0.65 ^a^	0.00 ± 1.55 ^a^	0.00 ± 1.52 ^a^	0.00 ± 19.38 ^abc^
Control (+) azadirachtin	0.88	0.00 ± 0.65 ^a^	−0.67 ± 1.85 ^a^	4.73 ± 3.75 ^a^	49.25 ± 11.89 ^cd^

^1^ Mortality percentage corrected by Abbott’s formula to correct the mortality percentage in the control was more than 5%. ^2^ Oviposition deterrent activity using Hematpoor formula: % OD = [(Cs − Ts)/Cs] × 100 where Cs and Ts represent the number of eggs laid on the control and in the treated container respectively. ^a–d^ Statistically significant differences by non-parametric Wilcoxon signed-rank test, *p* < 0.05.

**Table 6:** AChE inhibitory assay. IC_50_ values (mean ± standard deviation) and gradient concentration of terpene/terpenoid standards and *O. vulgare* subsp. *virens* EOs for AChE (n = 3).

Code	IC_50_ (mg/mL)	Gradient Concentration R^2^
Carvacrol	0.027 ± 0.000 ^a^	y = 46.56x + 123.14 0.981
Thymol	2.531 ± 0.515 ^d^	y = 22.56x + 45.52 0.936
Carvacrol/thymol (1:1)	0.070 ± 0.002 ^b^	y = 33.43x + 88.09 0.945
OVPEF (C)	0.086 ± 0.004 ^b^	y = 39.40x + 92.85 0.962
OVPS (T)	0.135 ± 0.003 ^c^	y = 35.40x + 80.77 0.945
OVLL (Mix)	0.109 ± 0.015 ^c^	y = 53.32x + 104.56 0.997
Control (+) galantamine hydrobromide	0.002 ± 0.000 ^e^	y= 35.33x + 146.62 0.984

IC_50_: Concentration of extract that inhibits 50% of AChE. ^a–e^ Statistically significant differences by ANOVA, Tukey’s test, *p* < 0.05.

**Table 7:** α- and β-GLU inhibitory assay. IC_50_ values (mean ± standard deviation) and gradient concentration of terpene/terpenoid standards and *O. vulgare* subsp. *virens* EOs for α- and β-GLU (n = 3).

Code	α-GLU		β-GLU	
	IC_50_ (mg/mL)	Gradient Concentration R^2^	IC_50_ (mg/mL)	Gradient Concentration R^2^
Carvacrol	109.34 ± 23.12 ^c^	y = 12.43x + 23.89 0.954	-	y = 9.86x + 23.04 0.966
Thymol	0.304 ± 0.025 ^a^	y = 38.36x + 70.47 0.972	-	y = 2.70x + 8.10 0.979
Carvacrol/thymol (1:1)	0.497 ± 0.091 ^ab^	y = 32.50x + 62.49 0.965	-	y = 5.66x + 20.23 0.990
OVPEF (C)	-	y = 8.89x + 17.39 0.991	-	y = 5.32x + 16.15 0.974
OVPS (T)	16.33 ± 8.15 ^b^	y = 18.73x + 33.83 0.976	-	y = 3.34x + 10.98 0.987
OVLL (mix)	90.25 ± 30.05 ^c^	y = 12.38x + 25.67 0.899	-	y = 6.41x + 12.18 0.981
Control (+) 1-DNJ	0.231 ± 0.005 ^a^	y = 34.11x + 71.96 0.955	0.036 ± 0.001 ^a^	y = 40.60x + 108.62 0.984

IC_50_: Concentration of extract that inhibits 50% of α- or β-GLU; ^a–c^ statistically significant differences by ANOVA, Tukey’s test, *p* < 0.05 (-): no inhibition of α- or β-GLU.

## Data Availability

The original contributions presented in this study are included in the article. Further inquiries can be directed to the corresponding author.

## Supplementary Materials

The following supporting information can be downloaded at: https://www.mdpi.com/article/10.3390/plants14193001/s1, Figure S1. GC-FID chromatogram for carvacrol chemotype OVPEF EO. Figure S2. GC-FID chromatogram for thymol chemotype OVPS EO. Figure S3. GC-FID chromatogram for thymol: carvacrol chemotype OVLL EO. Figure S4. GC-MS spectra for carvacrol chemotype OVPEF EO. Figure S5. GC-MS spectra for thymol chemotype OVPS EO. Figure S6: Biosynthetic pathway of thymol and carvacrol, adapted from Krause et al. (2021) [36]. Figure S7. Serial dilution for AChE inhibitory assay. Figure S8. Scheme for α-GLU and β-GLU inhibitory assay.
