# Supplementary material for: Origanum vulgare subsp. virens (Hoffmanns. & Link) Bonnier & Layens Essential Oils: Chemotypes and Bioactivity as Antifungal, Antifeeding and Enzyme Inhibitors
Source: Plants (Basel). 2025 Sep 28;14(19):3001. doi: 10.3390/plants14193001 (PMC12526278; doi:10.3390/plants14193001)
Supplement: Supplementary file 1 [file plants-14-03001-s001.zip › plants-3850842-supplementary.pdf]

Supplementary Material

Origanum vulgare subsp. *virens* (Hoffmanns. & Link) Bonnier & Layens: Chemotypes and Bioactivity as Antifungal, Antifeeding and Enzyme Inhibitors

Rui Ferreira[1], Mariana Martins[1],Vanessa Santos[1], Duarte Sardinha[2], Wilson Tavares[3], Samuel Sabina[4], Guacimara Espinel[4], Maria Carmo Barreto[3], Luísa Oliveira[5], Raimundo Cabrera[4], and Paula Castilho[1*]

[1] CQM - Centro de Química da Madeira, Universidade da Madeira, Campus da Penteada, 9020-105 Funchal, Portugal; rui.ferreira@staff.uma.pt; 2108020@student.uma.pt; vanessasilvasantos259@gmail.com; pcastilho@staff.uma.pt

[2] Laboratório de Qualidade Agrícola, Direção de Serviços dos Laboratórios Agrícolas e Agroalimentares, Direção Regional de Agricultura e Desenvolvimento Rural, Secretaria Regional de Agricultura e Desenvolvimento Rural, Caminho Municipal dos Caboucos nº 61, 9135-372 Camacha, Portugal; duarte.sardinha@madeira.gov.pt

[3] cE3c- Centre for Ecology, Evolution and Environmental Changes, Azorean Biodiversity Group, CHANGE – Global Change and Sustainability Institute, Faculty of Sciences and Technology, University of the Azores, Rua da Mãe de Deus, 9500-321 Ponta Delgada, Portugal; wilson.r.tavares@uac.pt; maria.cr.barreto@uac.pt

[4] Unidad de Fitopatología, Sección de Biología, Facultad de Ciencias, Universidad de La Laguna, Avda. Astrofísico Francisco Sánchez s/n, 38204 La Laguna, Tenerife, España; srodrisa@ull.edu.es; gespinel@ull.edu.es; rcabrera@ull.edu.es

[5] CBA - Biotechnology Centre of Azores, Faculty of Sciences and Technology, University of the Azores, Rua da Mãe de Deus, 9500-321 Ponta Delgada, Portugal; maria.lm.oliveira@uac.pt

* Correspondence: pcastilho@staff.uma.pt (Paula Castilho); Tel.: + 351 291 705 224

Table of contents

1. Gas Chromatography-Flame Ionization Detector (GC-FID) conditions 2
2. Gas Chromatography-Mass Spectrometry (GC-MS) conditions 2

Figure S1. GC-FID chromatogram for carvacrol chemotype OVPEF EO..2
Figure S2. GC-FID chromatogram for thymol chemotype OVPS EO..3
Figure S3. GC-FID chromatogram for thymol: carvacrol chemotype OVLL EO 3
Figure S4. GC-MS spectra for carvacrol chemotype OVPEF EO 3
Figure S5. GC-MS spectra for thymol chemotype OVPS EO 4
Figure S6: Biosynthetic pathway of thymol and carvacrol, adapted from Krause et al. (2021)......4
Figure S7. Serial dilution for AChE inhibitory assay 4
Figure S8. Scheme for α-GLU and β-GLU inhibitory assay...5

Equation S1. Insect antifeeding rate or refusal rate...5
Equation S2. Determination of oviposition deterrent activity 5
Equation S3. Determination of the inhibitory activity for AChE 5
Equation S4. Determination of the inhibitory activity for α - GLU 5

1. Gas Chromatography-Flame Ionization Detector (GC-FID) conditions

The EO characterization was performed using an Agilent 7890A gas chromatography (Agilent, Santa Clara, CA.) equipped with an autosampler Agilent 7693. The column used was a SPB™FA fused silica capillary column (30 m × 0.25 nm) with 0.20 µm film thickness (Supelco, Bellefonte, CA.) Helium was used as carrier gas with a flow rate of 800 µL/min. The GC oven followed a gradient temperature program, starting at 60 oC for 2 min, increased to 220 oC at 2 oC/min and held for 20 min; the total runtime was 102 min. Injector and FID detector temperatures were held at 250 oC. One µL of EO-hexane mixture was injected at a split ratio of 60:1, with a delay time of 4.0 min. Synthetic air and hydrogen were supplied to the FID detector at flow rates of 400 and 40 mL/min, respectively.

2. Gas Chromatography-Mass Spectrometry (GC-MS) conditions

The separation and qualitative analysis were carried out using an Agilent 6890N gas chromatograph (Agilent, Santa Clara, CA) equipped with an Agilent 5975 Inert Mass Selective Detector. An Agilent J&W HP-5 column, a nonpolar (5%-phenyl)-methyl polysiloxane column (300 × 0.32 mm I.D.) with a 0.25 µm film, was used. The oven temperature program followed the same profile as described for the GC-FID, with a constant helium column flow of 1.0 mL/min. For the MS conditions, the transfer line, quadrupole, and ionization source were maintained at 250°C, 150°C, and 230°C, respectively. Electron impact mass spectra were recorded at 70 eV, with an ionization current of 10 µA, and data acquisition was performed in scan mode (30–200 m/z).

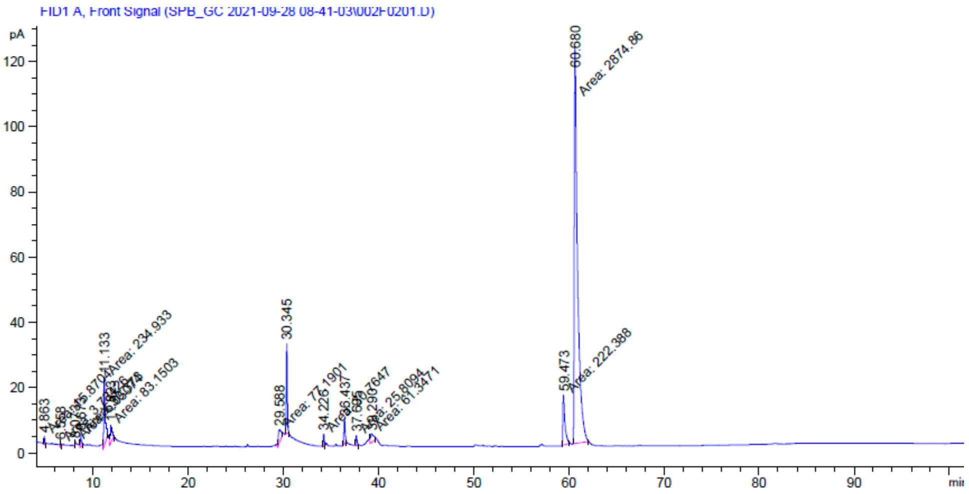

Figure S1. GC-FID chromatogram for carvacrol chemotype OVPEF EO

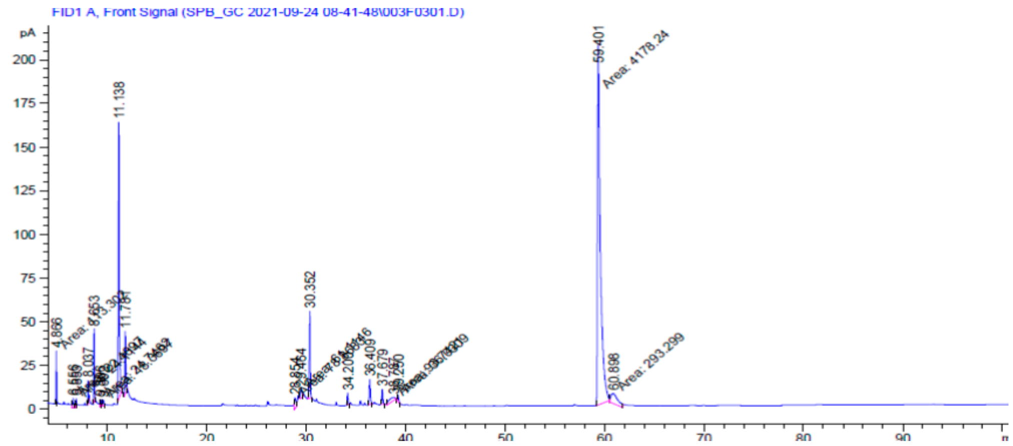

Figure S2. GC-FID chromatogram for thymol chemotype OVPS EO

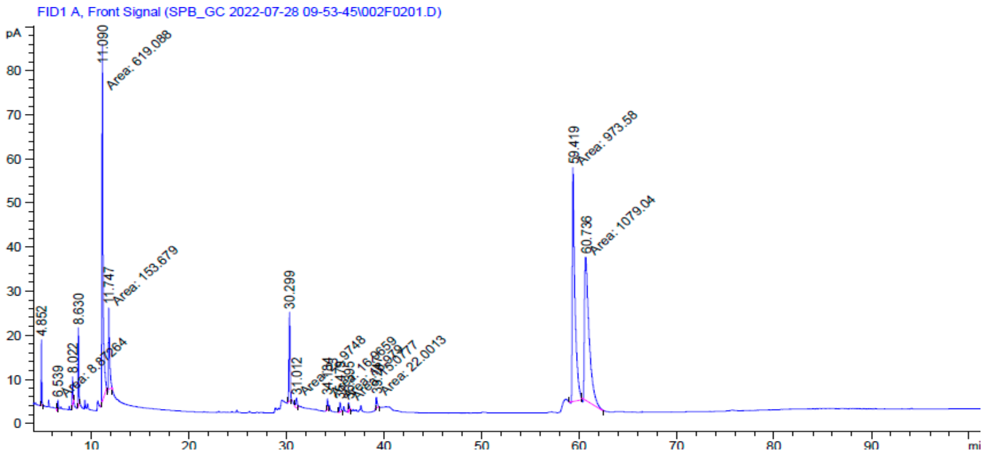

Figure S3. GC-FID chromatogram for thymol: carvacrol chemotype OVLL EO

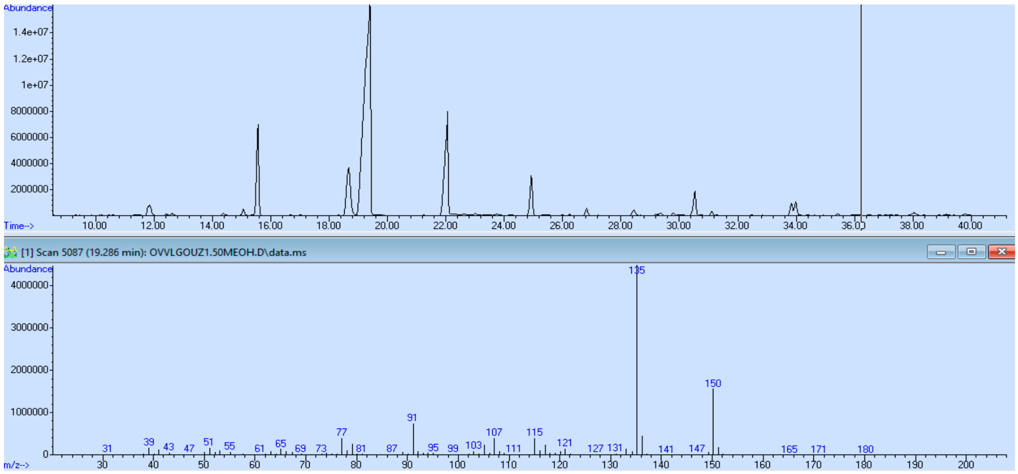

Figure S4. GC-MS spectra for carvacrol chemotype OVPEF EO

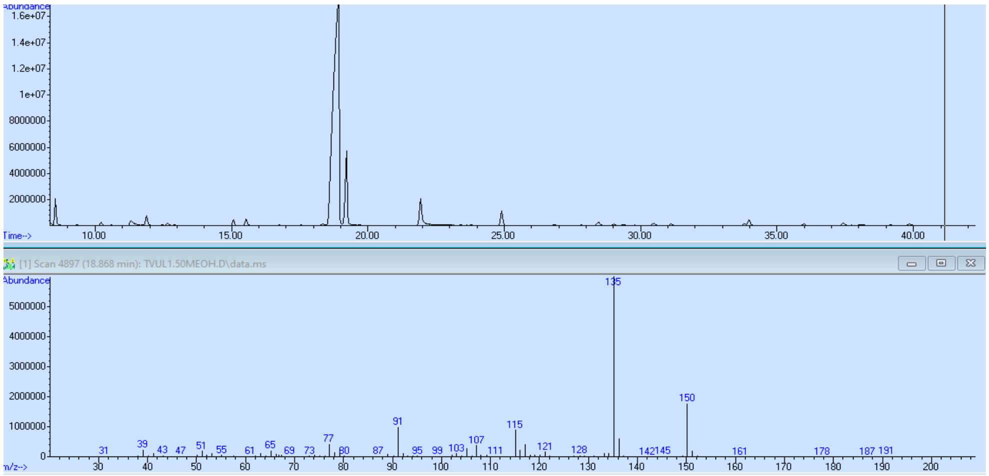

Figure S5. GC-MS spectra for thymol chemotype OVPS EO

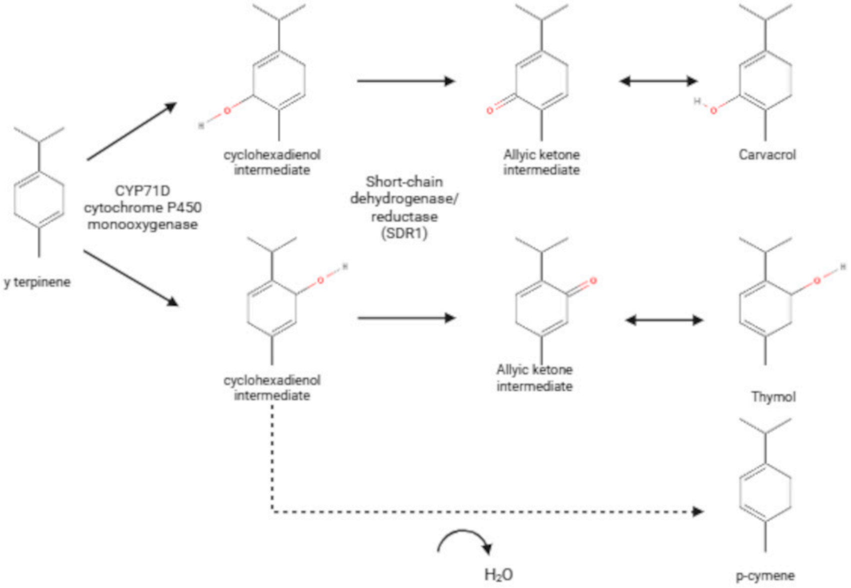

Figure S6: Biosynthetic pathway of thymol and carvacrol, adapted from Krause *et al.* (2021)

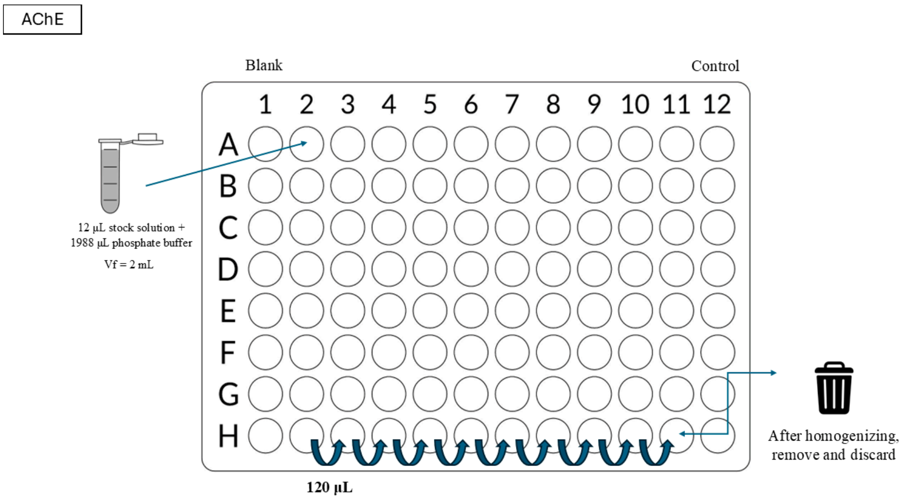

Figure S7. Serial dilution for AChE inhibitory assay

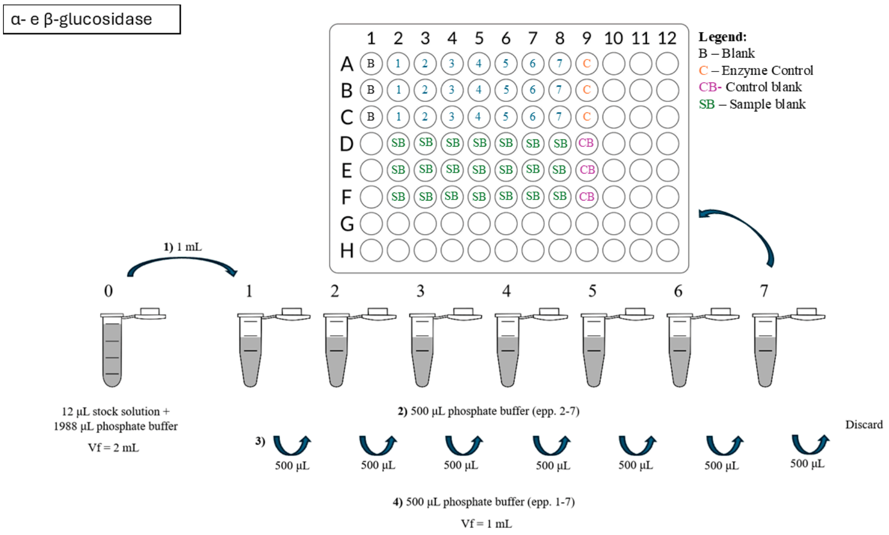

Figure S8. Scheme for α-GLU and β-GLU inhibitory assay

$$\%\ Refusal\ rate\ = \left(\frac{1 - \%\ consumption_{treated}}{\%\ consumption\ _{control\ ethanol}}\right) \times 100 \tag{1}$$

Equation S1. Insect antifeeding rate or refusal rate

$$\%\ \text{Oviposition deterrent activity}\ = \left(\frac{number\ eggs_{control} - number\ eggs_{treated}}{\text{number eggs}\ _{control}}\right) \times 100 \tag{2}$$

Equation S2. Determination of oviposition deterrent activity

$$\%\ Inhibition_{AChE}\ = \left(100 - \frac{1 - v_{sample}}{\text{v}\ _{control}}\right) \times 100 \tag{3}$$

Equation S3. Determination of the inhibitory activity for AChE

$$\%\ Inhibition_{\alpha-GLU}\ = \left(\frac{(A_{control} - A_{control\ blank}) - (A_{sample} - A_{sample\ blank})}{(A\ _{control} - A_{control\ blank})}\right) \times 100 \tag{4}$$

Equation S4. Determination of the inhibitory activity for α - GLU
